# Supplementary material for: Identification of photoperiod-induced specific miRNAs in the adrenal glands of Sunite sheep (Ovis aries)
Source: Front Vet Sci. 2022 Jul 22;9:888207. doi: 10.3389/fvets.2022.888207 (PMC9354845; doi:10.3389/fvets.2022.888207)
Supplement: Supplementary file 1 [file Data_Sheet_1.ZIP › Supplementary file/miRDeep2 file.zip!/miRDeep2 file/Novel_Structure/SP42A3/Novel_470.pdf]

Provisional ID	:	NC_019484.2_32650
Score total	:	27.7
Score for star read(s)	:	3.9
Score for read counts	:	20.5
Score for mfe	:	1.7
Score for randfold	:	1.6
Score for cons. seed	:	
Total read count	:	52
Mature read count	:	47
Loop read count	:	0
Star read count	:	5

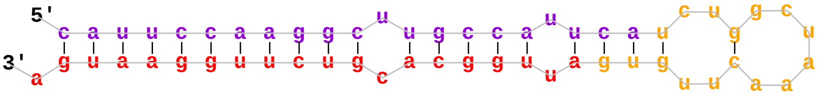

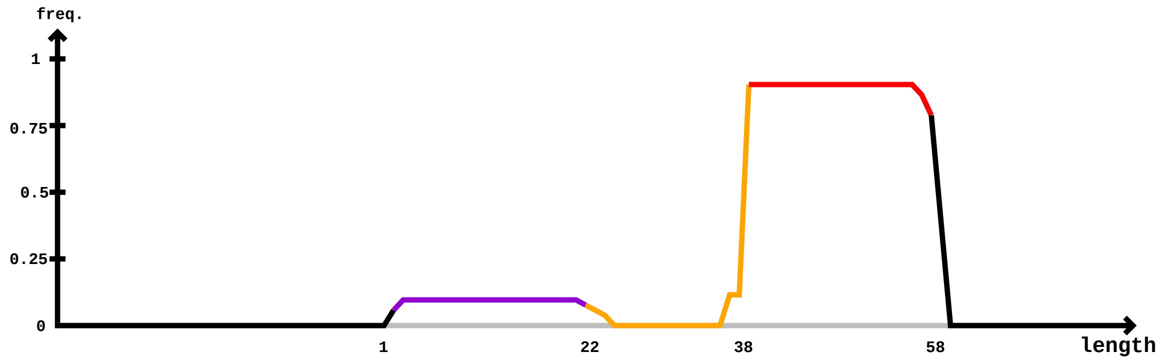

Star **Mature**

```
5'- uugccaccuucagcgugucugugugcuguaccccauuccaaggcuugccauucaucuggcuaaacuugugauuggcacgucuuggaaugaaguaacguacaacaaugaug   -3'    obs
    uugccaccuucagcgugucugugugcuguaccccauuccaaggcuugccauucaucuggcuaaacuugugauuggcacgucuuggaaugaaguaacguacaacaaugaug          exp
    ..((........))..((((((((((..(((..(((((((((((.(((((.((((..(......)..)))).))))).)))))))))))..)))..)))).)))..))).   reads    mm    sample
    .................................cauuccaaggcuugccauuc.........................................................   1        0     seq
    .................................cauuccaaggcuugccauuca........................................................   2        0     seq
    ..................................auuccaaggcuugccauucauc......................................................   2        0     seq
    ....................................................................ugauuggcacgucuuggaau......................   1        0     seq
    ....................................................................ugauuggcacgucuuggaaug.....................   1        0     seq
    ....................................................................ugauuggcacgucuuggaauga....................   4        0     seq
    ......................................................................auuggcacgucuuggaau......................   1        0     seq
    ......................................................................auuggcacgucuuggaaug.....................   3        0     seq
    ......................................................................auuggcacgucuuggaauga....................   26       0     seq
    ......................................................................auuggcacgucuuggaaugaa...................   11       0     seq
```
